# Helping as a Concurrent Activity: How Students Engage in Small Groups While Pursuing Classroom Tasks

**DOI:** 10.3389/fpsyg.2021.784906

**Published:** 2022-01-13

**Authors:** Denise Wakke, Vivien Heller

**Affiliations:** Department of German Studies, School of Humanities and Cultural Studies, University of Wuppertal, Wuppertal, Germany

**Keywords:** helping, classroom interaction, multiactivity, dual involvements, space, moral commitment, multimodal interaction analysis

## Abstract

This study examines interactions in which students help each other with their learning during classroom instruction, forming groups in the process. From a conversation analytic perspective, helping is assumed to be a sequentially organized activity jointly accomplished by the participants. As an activity that proceeds alongside other ongoing classroom activities, helping can be conceived as part of a *multiactivity* that poses students with multi-faceted interactional and moral challenges. While previous research on helping in educational contexts has primarily focused on the influence of helping on learning outcomes and social dynamics in helping interactions, the present study investigates how students cope with the intricacies of moral commitments inherent in helping as a concurrent activity. The aim of this paper is two-fold. First, we aim to elaborate on how students’ dual involvements – i.e., their involvement in classroom activities while simultaneously providing help – manifest in the ways in which groups are constituted, maintained, and dissolved. The analyses reveal that both the compatibility of helping with the activity already in progress as well as the students’ problem definition are consequential for the sequential and bodily-spatial unfolding of the help interaction, inducing different arrangements that constitute a continuum, at each end of which there is a dominant orientation toward the *shared space of helping* or toward the *individual/collective space*. Furthermore, from a methodological perspective, our study aims to demonstrate the extent to which multimodal interaction analysis is applicable when examining naturally occurring groups, in this case, in interactive processes of helping. The study is based on a data corpus that comprises video recordings of mathematics and German lessons from two fifth-grade classrooms.

## Introduction

Students helping each other is an ordinary activity in the classroom, and educational research has emphasized its relevance for self-regulated learning (Newman, 1994). At the same time, it is often assumed that students’ help processes need to be improved in order to promote learning. To find out how to increase the effectiveness of helping for learning, internal and external factors influencing help, such as cognitive and affective abilities (Newman and Goldin, 1990; Karabenick and Knapp, 1991; Tobias and Everson, 2002), the quality of group interactions and group dynamics (Kempler and Linnenbrink, 2006; Webb et al., 2006), and the social composition of groups (Oswald and Krappmann, 1988; Zornemann, 1999; Campana Schleusener, 2012; Wagener, 2014) were identified. In these studies, the actual interactive contexts and moral orders in which spontaneous help is given in the classroom have so far remained unconsidered. In our data, helping occurs during individual work periods and whole-group discussions and competes with the students’ commitment to working on an assignment, paying attention, and/or contributing to an ongoing multiparty discourse. The fact that helping in the classroom is often a *concurrent activity* poses complex interactive and moral challenges for the participants and the way they constitute a group while helping. This article reconstructs these challenges as well as the practices students draw on to tackle them. The background is the assumption that these interactive and moral orders cannot be ignored and that interventions that seek to improve the learning potential of help interactions must take them into account.

Drawing on conversation analytic work, we understand helping as a jointly accomplished and sequentially organized activity (Svahn and Melander Bowden, 2019) for which participants employ various discursive practices and communicative resources. Most importantly, we account for the fact that helping among students is often part of a *multiactivity* (Mondada, 2011; Haddington et al., 2014), such as when students are simultaneously expected to help a classmate *and* to participate in a whole-class discussion or to work on an individual task. Based on the works of Goffman (1963, 1964) and his notions on the structuring of social gatherings, this means that in the context of helping, the students initiate a *face engagement* or *encounter*, which is defined as an association “of two or more participants in a situation joining each other openly in maintaining a single focus of cognitive and visual attention – what is sensed as a single *mutual activity*, entailing preferential communication rights” (Goffman, 1963, p. 89). A characteristic of an encounter is that the participants feel a moral responsibility or “we-rationale” for their actions (ibid., p. 97f.). At the same time, the students are part of the classroom *gathering* (Goffman, 1964) that, due to its institutional purpose, involves focusing on and completing collective or individual assignments. Therefore, when students respond to requests for help from their classmates during activities, they must simultaneously manage two involvements (Goffman, 1963; Peräkylä et al., 2021) and two moral commitments (Clark, 2006; Raymond and Lerner, 2014): the obligation to help and the obligation to work on the teacher’s assignment. Our interest is in how students cope with these *moral intricacies* inherent in helping and in the varied and complex tasks they must manage when seeking and providing help as a *concurrent activity*. Specifically, the focus is on the following analytical questions: (i) How do students sequentially organize help interactions and (ii) what bodily-spatial arrangements do they make for engaging in helping as a concurrent activity in the classroom? On this basis, we discuss how their dual involvements manifest in the ways in which groups are constituted, maintained, and dissolved. We address these questions by investigating help sequences between fifth graders in naturally occurring interactions within different classrooms, during individual work periods and whole class discussions.

Drawing on multimodal interaction analysis, we describe different ways in which participants, depending on their *definition of the problem* as being more or less complex, insert help into concurrent courses of actions and balance two competing moral commitments. How these commitments are balanced is reflected in the practices participants draw on: help can be either achieved by rather short adjacency pairs (Schegloff and Sacks, 1973) or more extended discourse units (Wald, 1978). Participants’ prioritization of a certain commitment also becomes manifest in the bodily-spatial arrangements they create while helping. Building upon work by Ciolek and Kendon (1980), we conceptualize different spaces that participants create and orient to: the *individual space of task processing*, the *collective space* of the (teacher-led) classroom activity (Schmitt, 2013), and the *shared space of helping.* We propose that the orientation to the individual/collective space and the shared space of helping constitute a continuum, at the ends of which students either heavily commit to helping or remain primarily focused on the individual task or the plenary activity. The different orientations can also be considered key resources for handling multiple moral commitments and to create different degrees of “withness” (Ciolek and Kendon, 1980, p. 244) and involvement with the group. At the same time, we trace that orientations are also subject to negotiation and can change in the course of the activity.

We begin by discussing educational and linguistic perspectives on helping in the classroom. Based on a conceptualization of helping as a concurrent activity, we explicate how we utilized multimodal interaction analysis to uncover sequential, spatial, and moral orders of helping. In our analysis, we present two sample cases of helping activities that differ in how complex students define the problem and whether they judge helping to be compatible with the activity already in progress. The analysis addresses different sequential and bodily spatial arrangements when helping and illustrates how these arrangements are consequential for the constitution of the group. The paper concludes with a discussion of the findings and the methodological approach.

## Theoretical Frame

### Helping in Educational Settings

Previous research on helping in educational settings can be categorized into two major strands of research that approach helping either more quantitatively, from the perspective of educational psychology (e.g., Nelson-Le Gall and Gumerman, 1984; Newman, 1990, 2002; Newman and Goldin, 1990; Ryan and Shim, 2012; Schenke et al., 2015), or more qualitatively, from the domain of educational science (e.g., Oswald and Krappmann, 1988; Kauke and Auhagen, 1996; Zornemann, 1999; Campana Schleusener, 2012; Koole, 2012; Melander, 2012; Wagener, 2014; Svahn and Melander Bowden, 2019). While works from educational psychology tend to focus on the individual, those from educational science focus on helping as an interaction.

In the field of educational psychology, a vast number of studies have dealt with help-seeking as an “adaptive strategy of self-regulated learning” (Newman, 1994, p. 285) and with effective help behavior (e.g., Webb and Mastergeorge, 2003), i.e., conducive to learning, that correlates positively with school success (Webb and Mastergeorge, 2003; Ryan et al., 2005; Ryan and Shim, 2012; Schenke et al., 2015). These investigations have determined internal and external factors influencing individual help(-seeking) behavior. Internal factors include cognitive (Karabenick and Knapp, 1991; Tobias and Everson, 2002; Tobias, 2006), affective-emotional (Newman and Goldin, 1990), and social competencies, as well as individual academic achievements (Newman, 1990; Newman and Goldin, 1990; Ryan et al., 1997) and achievement goal orientations (Karabenick, 2004; Karabenick and Newman, 2009, 2010). These studies illustrate that highly developed metacognitive skills and a positive self-concept contribute to effective, instrumental help-seeking (Nelson-Le Gall, 1985, p. 67), whereas help-seeking is impeded by low cognitive skills and self-doubt (e.g., Karabenick and Berger, 2013). Aspects such as the classroom climate (Karabenick and Newman, 2009), the quality of group interaction and group dynamics in cooperative learning settings (e.g., Kempler and Linnenbrink, 2006; Webb et al., 2006), and familial and instructional socialization processes (Newman, 2000) have been investigated as external factors. Karabenick and Berger (2013, p. 239ff.) have developed a model that depicts the help-seeking process in an ideal-typical way and involves a series of stages and decision points, but they have focused primarily on the individual’s cognitive mechanisms. Drawing on extensive research efforts, they also worked out the competencies required for the accomplishment of this process and designated cognitive, affective-emotional, contextual, and social skills (ibid., p. 245ff.). Although educational psychological research points out that “seeking help […] involves others” (ibid., p. 238), the significance of communicative and interactional competencies is mentioned only incidentally (cf. Nelson-Le Gall, 1985, p. 76–77; Newman, 2000, p. 352).

These primarily quantitatively designed studies stand in contrast to educational science studies, which investigate helping as an interpersonal process, using predominantly qualitative approaches. This second strand of research can again be further subdivided into two branches. On the one hand, some studies focus on different varieties of interactions regarding social features of the process of helping and describe, among other things, participant constellations (e.g., mixed-age, age-homogeneous, mixed-ability, and friendly vs. non-friendly dyads; Kauke and Auhagen, 1996; Zornemann, 1999; Benkmann, 2004; Campana Schleusener, 2012), courses of interaction (e.g., Oswald and Krappmann, 1988; Zornemann, 1999; Wagener, 2014), and help actions (Campana Schleusener, 2012; Wagener, 2014) in different types of classroom settings, such as mixed-age groups (e.g., Campana Schleusener, 2012; Wagener, 2014), age-homogeneous groups (e.g., Oswald and Krappmann, 1988; Zornemann, 1999), or inclusive learning groups (e.g., Beaumont, 1999; Benkmann, 2002, 2004). Wagener’s (2014) work (2014, p. 257–263) regarding the interactional requirements of learning-related helping is particularly relevant. Based on observation protocols, she traces the interaction process of mutual helping and reconstructs four phases. Hence, the participants must first (1) initiate the interaction. Subsequently, they must (2) negotiate the (non-)occurrence of the help, (3) accomplish the activity of helping, and finally (4) agree on the consensual completion of the help process. This reconstruction provides initial insights into the sequential unfolding of helping-in-interaction and already suggests the interactive and also discursive complexity of the activity. The sequential organization of helping is also examined by Svahn and Melander Bowden (2019). Based on video recorded interactions between a tutor and group of students in a mathematical homework support setting, these authors reveal that helping involves a series of interactive jobs that participants engage in together, including (1) initiating a help request, (2) localizing the problematic assignment, (3) presenting the problem, and (4) providing instructions or explanations. Through their multimodally framed analysis, Svahn and Melander Bowden further expose the importance of “participants’ use of gesture and other forms of bodily activity to establish mutual orientation to particular objects within the local environment” (ibid., p. 18) and elaborate on the crucial role of material resources, which, as epistemic resources (ibid., p. 18), significantly contribute to the determination of the problematic issues. Through these detailed analyses, they illustrate that the process of helping is highly complex on an interactional level and is always accomplished through the participation of and cooperation between all interactants.

The state of research outlined in this section shows that helping in educational settings has been investigated from different perspectives. Research from the field of educational psychology emphasizes the central role of helping in individual learning, while interactional approaches in educational science emphasize social dynamics, revealing that help interactions and actions in educational contexts are highly diverse. Beyond that, conversation analytic studies focus specifically on interactive characteristics of helping and examine it as an interactional and embodied achievement of all participants. These works demonstrate that the activity of helping is challenging and quite delicate for participants at many levels. In the following section we now shed light on helping from a linguistic perspective.

### Helping and Related Practices From the Perspective of Linguistics

In the first place, the notion of *helping* is a lay term or ethnocategory which is based on the understanding that the help recipient is supported in his or her actions by the helper relieving the recipient of difficult or problematic actions or action steps that overburden him or her. This help can be provided both verbally and non-verbally, through talk and/or practical acting (Pick and Scarvaglieri, 2019, p. 26). Examples of this would be when a clerk at the department of public welfare explains to the client where to find the missing document for his/her application and what steps to take next, or the educator takes over tying the shoes of a child who has not yet learned to do so (ibid., p.26). Depending on the situational context, helping can fulfill different functions: On the one hand, it can aim at enabling the help recipient to accomplish the corresponding action himself/herself (as with the clerk’s explanation), or, on the other hand, it can more or less completely replace the recipient’s action without promoting his/her own agency (as with the educator tying the shoes). Regardless of these different functions, the helping person is assumed to have a certain knowledge, skill (ibid., p. 7) or possession (e.g., Wagener, 2014; Wakke, 2021).

In discourse analysis, the term *helping* has been used primarily in the context of advising as a generic term for different advisory forms of action (cf. also Pick, 2017, p. 430f.). An examination of helping as a linguistic and interactional phenomenon in its own right, however, has remained unconsidered for a long time and has only recently begun. Pick and Scarvaglieri (2019), for example, focus on a conceptualization of *helping in and through language* (ibid., p. 19) that follows the tradition of discourse analysis, describing it as a complex activity that goes beyond a single action, comprises several sub-actions that need to be managed interactionally, and is in turn embedded in superordinate actions. Kendrick and Drew (2016) also examine, in a conversation analytic way, helping and its recruitment in mundane interactions, but use the term of *assistance* (ibid. Drew and Kendrick, 2018; Kendrick, 2021), which is largely synonymous with the term of *helping* that we use. In the vein of conversation analysis, they conceive *assistance* and its recruitment as “a basic social organizational problem for which participants have practiced solutions” (Kendrick and Drew, 2016, p. 2; see also Kendrick, 2021, p. 79).

Help interactions can be organized both as longer discourse units (Wald, 1978) or as adjacency pairs (Schegloff and Sacks, 1973). For longer help interactions, discursive practices such as *advising* and *explaining* are typical. What characterizes these discursive practices is that they are organized globally on a sequential level and therefore exhibit a certain complexity. We only discuss explaining in this paper because this practice is particularly relevant to our analysis. On a structural level, explaining includes five jobs that have to be successively accomplished by the interactants, thus providing the sequential organization of and an orderliness to the interactively constituted explanation process. Within these five jobs, the interactants are required to establish topical relevance; constitute an explanandum; explicate procedural, conceptual and/or causal relations; organize the closing; and transition to the interrupted or a newly begun activity (cf. Morek, 2012; Heller, 2016; Quasthoff et al., 2017). To jointly accomplish these jobs, the participants use various devices, which are realized through a range of linguistic, prosodic, and non-verbal resources. Morek further states that explanations “are usually linguistically complex in the sense that they involve the construction of coherently structured units above the sentence level” (2015, p. 239f.). Similarly, advising ranges from more locally organized, sentence-based advice to globally organized, complex advice sequences (Heritage and Sefi, 1992; Locher and Limberg, 2012). Consequently, the discursive practices are not only sequentially complex, but also linguistically demanding.

In contrast, as Kendrick and Drew (2016, p. 10) already pointed out, helping can also be organized locally through *adjacency pairs* (Schegloff and Sacks, 1973; Schegloff, 2007, p. 13; see also Kendrick, 2021). The sequential organization of these shorter units arises via so-called local conditional relevancies; i.e., normative expectancies between utterances (ibid., p. 13, 20). Typical adjacency pairs in the context of helping are, for instance, requests for information – answer, offer – accept/decline, and request – grant/decline. Therefore, at the linguistic level, helping is highly diverse and can be realized with different practices of varying complexity, adopting different forms. Like educational research, linguistic research has not yet investigated students helping each other as part of multiactivities. However, these are likely to represent a common context of helping in everyday school life. We suggest that everyday manifestations and organizational forms of helping between students in the classroom can only be understood more accurately if helping is understood to be part of a multiactivity. In the following section, we will introduce this concept, which is central to our analysis, and explain our analytical approach, which takes sequential, moral, and spatial orders of helping into account.

## Materials and Methods

### Analytical Concepts

In our data, spontaneously occurring help interactions among students are usually characterized by the fact that helping is not the sole activity, but is embedded in an activity which has previously begun, such as the individual processing of a task or a whole group discussion. Conversation analytic research refers to such involvements in concurrent courses of action as “multiactivity” (Mondada, 2011; Haddington et al., 2014). This form of interaction is typical of many contexts, including classroom interaction. While classroom interaction is often conceptualized as a single activity with only two participating parties – the teacher and the class – on closer examination, it turns out to be characterized by a multitude of “parallel activities” (Koole, 2007) that may be initiated, tolerated, or overlooked by the teacher.

In conversation analysis, multiactivity is analyzed as an interactively accomplished phenomenon (Haddington et al., 2014) that draws on the participants’ ability to divide their “involvements,” i.e., their concerted attention to some activity at hand” (Goffman, 1963, p. 43). Rather than focusing on individual cognitive processing of multiple tasks (multitasking), this line of research is interested in the ways in which participants engage in and coordinate multiple activities and participation frameworks (Goodwin, 1981); i.e., participants’ bodily arrangements that create an environment for mutual attention and perception and are thus fundamental to joint meaning making. To examine helping in the classroom as a multiactivity, our analytical approach addresses three dimensions: the sequential, moral, and spatial dimension.

As to the *sequential dimension* of multiple activities, participants need to deal with the practical problem of coordinating the simultaneous demands that each activity poses with regard to its sequential organization and the use of communicative resources. Multiactivities can entail either two interactive projects or one individual and one interactive project. The concurrent courses of action can be organized simultaneously, such as when handing an object over to a customer while telling other customers a story (Raymond and Lerner, 2014). In cases like this, most of the communicative resources can be divided between the two activities. In the example with the customers, while verbal resources are reserved for the storytelling, the hands are used for handing over the change. The fact that gaze is used, even if briefly, for both activities indicates that a competition of resources may occur. If two strands of action are not compatible due to a competition of resources, they will be organized successively; i.e., one activity can be embedded in another one which is temporarily paused and later resumed (e.g., Haddington et al., 2014). Multiactivities can also involve multiple dis-engagements and re-engagements. For example, this can be observed in class during individual work. Szymanski (1999) examines how third graders sitting at group desks deal with the challenge of engaging in talk with another child who is currently working on an individual reading or writing assignment. The practices for re-engaging with fellow students include sequence-initiating actions such as questions, announcements, and noticings. Dis-engagements, in contrast, are achieved by visibly and audibly continuing with the individual task. The examples show that different temporal orders can be established for the accomplishment of multiple activities and that multimodal resources play a central role in this process. In our analysis, we trace the sequential order established in helping during individual tasks and plenary announcements by the teacher.

The *moral dimension* of helping-within-multiactivities implies that multiactivities bring along the need to establish a hierarchy between competing activities. While one activity is treated as a priority (Mondada, 2014b) or main involvement (Goffman, 1963), the other activity assumes the status of a side involvement (Goffman, 1963), less important or postponable action (Deppermann, 2014). For example, Raymond and Lerner (2014) describe two forms of “adjusting actions”: suspending (e.g., suspending the act of seasoning one’s own food in favor of fulfilling a concurrent request to serve food) and retarding (e.g., delaying a payment routine to simultaneously remind the customer to take the coffee with them), that enable different relations between two relevant courses of actions. We argue that this relation is not just a matter of sequencing but is also related to moral orders: When members engage in activities (or are in charge of the execution of certain actions qua role, e.g., a seller being responsible for a purchase transaction), they commit themselves (Clark, 2006; Raymond and Lerner, 2014) to the activity and the purpose it serves to fulfill (Clark, 2006; Raymond and Lerner, 2014). Committing oneself to an activity means holding oneself as morally responsible for it. The simultaneous performance of multiple activities presents participants with the challenge of managing these responsibilities. This problem comes to a head in the case of student help interactions in the classroom, because there is both a strong moral obligation to help and a strong obligation to follow the teacher-directed course of actions. In our analysis, we describe the practices students draw on to tackle this dilemma.

The *spatial dimension* of helping-within-multiactivities refers to the bodily spatial arrangements that participants create to pursue two courses of action. A common arrangement, especially for small gatherings, is the F-formation (Kendon, 1990); i.e., a spatial configuration with which people establish “an overlap of their transactional segments […], enabling them to use these segments as a joint interaction space” (Ciolek and Kendon, 1980, p. 241; cf. Hausendorf, 2013; Mondada, 2013), which is also called “o-space” (Ciolek and Kendon, 1980, p. 243). Ciolek and Kendon describe different F-formations that are open or more closed. When people come to stand or sit vis-à-vis and face each other directly, they constitute a closed arrangement, because they shield their encounter from other participants. In contrast, when people stand or sit side-by-side, facing in the same direction but still having full access to each other’s transactional segment, they create an open arrangement with fuller access to the immediate environment. The authors argue that the different shapes are not only related to the physical environment and the type of activity, but also document different degrees of involvement: “By standing close or far, by orienting fully or only partly to one another, participants may express the extent to which they are involved in the encounter at hand that is, whether their attention is wholly or only partly taken up with it” (ibid., p. 237f.). Thus, the different shapes can be also considered key resources to create different degrees of interactional and social “withness” (ibid., p. 244) and involvement in a group activity. In multiactivities, participants manage two interactional spaces (or an individual and a joint transactional segment). Our analysis describes the context-specific forms this takes in the classroom, because a collective interactional space (Schmitt, 2013) is usually established here to which participants must somehow relate when constructing parallel dyadic interactional spaces.

Methodologically, our analytical approach is informed by multimodal interaction analysis (Goodwin, 2000; Streeck et al., 2011; Mondada, 2014a), a micro-detailed approach to the study of human interaction and sense-making in diverse social and material environments. Drawing on methods and insights developed by conversation analysis (Sacks, 1995; Sidnell and Stivers, 2013) and context analysis (Ciolek and Kendon, 1980; Kendon, 1990), this approach allows for the exploration of the students’ practices for constituting, maintaining, and dissolving groups when engaging in help through examining a broad range of spatial, material, and multimodal resources: materials on the students’ desk, body posture, gaze, gesture, facial expression, prosody, and talk.

### Data Materials

Our study is based on naturalistic data consisting of video recordings of 22 German and mathematics lessons in two fifth-grade classrooms, one class each from a Gymnasium and a comprehensive school^1^. The seating arrangements in the classrooms varied. In the comprehensive school class, the students’ desks are organized in clusters; at the Gymnasium, students were seated at tables aligned parallel or perpendicular to the blackboard. The lessons were recorded with a total of four cameras, one following the teacher (who wore a wireless microphone), a second one recording the entire classroom from the front of the class (total view), and two more cameras capturing groups of students (with additional voice recorders on the students’ desks).

For the data preparation, all “candidates” for help interactions among students were annotated in MAXQDA. In order to be able to describe everyday help interactions in their multi-faceted nature, no narrow criteria were applied in the annotation. Instead, all phases of instruction (introduction, individual work, and class discussions) were examined. With one exception, the four teachers did not explicitly ask students to help each other. All of the help interactions captured were therefore spontaneously initiated by the students. The objects of the help interactions were varied and ranged from requests for class materials to questions about the spelling of a word and other complex content-related issues. Overall, a total of 143 sequences were identified in which a request or offer for help occurred. They cover both self-initiated and other-initiated help interactions.

The total of 143 sequences were transcribed according to the notation conventions of “Gesprächsanalytisches Transkriptionssystem 2” (GAT 2, cf. Selting et al., 2011). This method of transcription captures not merely what was said, but also how and when something was said. It also allows researchers to register multimodal actions, such as material arrangements, changes in body posture, gestures, and facial expressions in terms of their temporality and their alignment with the verbal utterance. GAT 2 transcripts thus enable the researcher to analyze the intricate actions that characterize help interactions and the ways in which they are coordinated with other activities (e.g., writing, solving math problems). Anonymized still images were integrated into the transcripts to illustrate relevant spatial arrangements and embodied actions in the transcript.

## Analysis: Helping as a Concurrent Activity – Handling Multiple Courses of Action and Moral Commitments

Help activities can be set relevant at almost any time in class, such as in individual work phases in which students are requested to work on a pre-determined task, within phase changes, and in plenary activities. Regardless as to when interactive negotiations of helping emerge among students, helping and the concomitant group formation process constitute an activity competing against the already ongoing course of action, so the students are in a state of having to manage these competing activities and the moral commitments they entail. Using two examples, this section reconstructs how the students’ problem definition as complex (Section “Helping in Handling a Problem Defined by the Students as Complex”) or uncomplex (Section “Helping in Handling a Problem Defined by the Students as Uncomplex”) affects the students’ organization of the help interaction and their handling of dual moral commitments. In order to expose how students manage their moral commitments to both helping and individual/plenary tasks, we will first address the sequential organization and then the bodily spatial arrangements. Finally, we bring the findings together and discuss the extent to which different arrangements are consequential for the constitution of the group (Section “Summary”).

### Helping in Handling a Problem Defined by the Students as Complex

The first sequence (Excerpt 1) is taken from a German lesson at the comprehensive school, in which the students are involved in an individual task and are assigned to type a response letter on their laptops. During this individual work phase, Paula encounters a spelling problem while writing the term “Lieblingsfächer” (favorite subject), and approaches Tijen. Note that in their negotiation, Paula and Tijen interactively define the problem to be solved as highly complex.

**EXCERPT 1:**
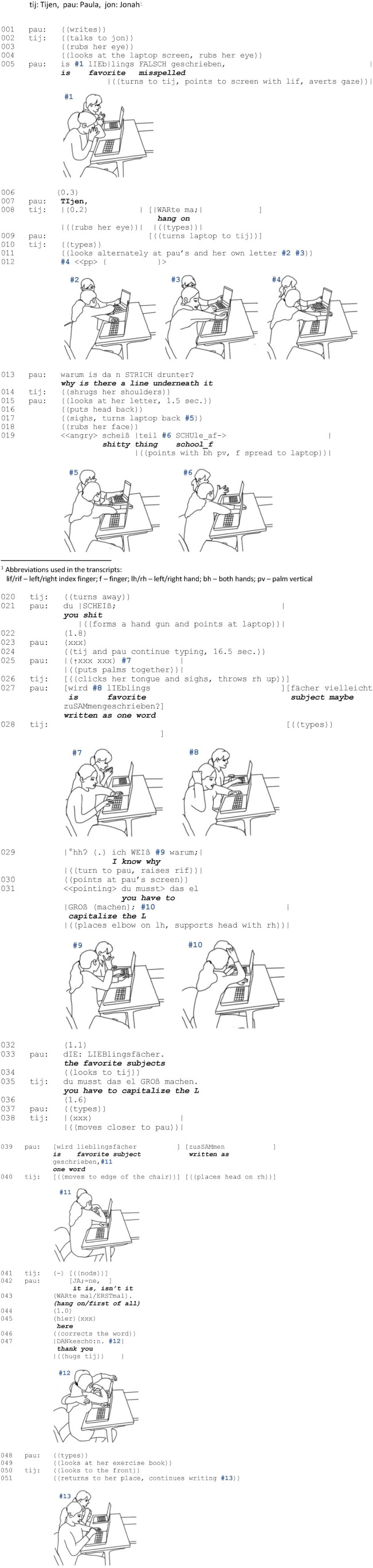
3DU_120907_15_2.

#### Sequential Organization

At the beginning of the sequence, Tijen indicates that she is not complying with the teacher’s previously imposed commitment to complete the task. Instead, she talks to Jonah (line 002) and then rubs her eye, which seems to be causing problems (lines 003f.). In the context of a copresent student group, Szymanski (1999) refers to such activities as pre-re-engagement actions preceding a re-engagement since “[copresent] individuals may make visible a place for re-engaging talk by discontinuing individual work” (ibid., p. 8). Paula takes this opportunity and initiates a help interaction by raising a help request in the form of a polar question while detaching from typing the letter (line 005). By doing so, she indicates epistemic uncertainty and constitutes an explanandum, so that over the further course of interaction, an explanatory discourse unit is to be expected (Morek, 2012, p. 68f.). Since Tijen does not instantly respond to this question, Paula poses a summons (Schegloff, 1968), to which Tijen replies metadiscursively (“hang on,” line 008) in order to clarify that the action in progress, i.e., the processing of the letter, which she resumed shortly after the summons, still has to be continued until a caesura. In doing so, she establishes a hierarchy between the two courses of action, constituting typing the letter as the *main activity* and helping as the *side* (Mondada, 2014b, p. 46) and *interjected activity* (Raymond and Lerner, 2014). After reaching the caesura, Tijen pauses the main course of action, turns to Paula’s problem, and Paula re-constitutes the previously established explanandum in a modified form (line 013). Tijen then displays a lack of knowledge by shrugging her shoulders, so that the transition into the expectable explanation fails (line 014).

Subsequently, this failure is followed by a series of verbal and embodied affect displays. Paula curses and produces response cries (Goffman, 1978). Furthermore, she puts her head back, rubs her face, and forms her hands into the shape of a gun, with which she shoots at the laptop in temporal alignment to cursing. These displays are similar to Kendrick and Drew’s (2016) trouble alerts for recruiting a potential helper, but here serve less to initiate a help sequence (ibid., p. 7). Rather, because of their sequential position, the affect displays serve as resources to keep the recipient in line. Although Paula does not immediately succeed at this and Tijen re-engages in the interaction only after Paula too has independently returned to her work (line 024), based on Goffman (1978), we assume that this form of public self-talk or outburst is directed at Tijen as a copresent other due to its fundamental display character. Note, however, that its inherent conditional relevance is rather weak, and therefore, it is up to Tijen to pursue the request for help or not (ibid., p. 794, 799). By using this practice of indirect, barely binding mobilization of help, Paula is taking into account the facts that Tijen is simultaneously pursuing another commitment and that she is not entitled to Tijen’s help. After Tijen does not respond to these affect displays and Paula continues working, Paula re-initiates the help interaction in line 027 by proposing an explanation and subjecting it to negotiation. Immediately thereafter, Tijen also expresses a change of state referring to Paula’s problem (line 029) and provides an alternative explanation in the form of minimal instruction (line 031). From this point on, the established hierarchy shifts, the former main activity is put on hold for a longer and interactively quite intense duration, and the help process is temporarily constituted as the new main activity.

**FIGURE 1:**
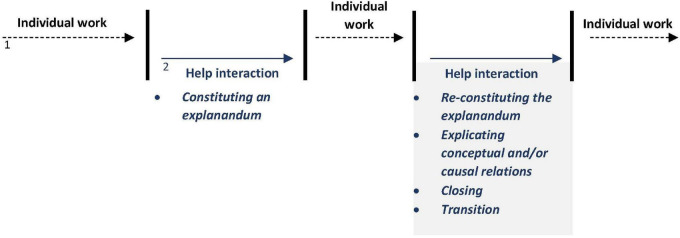
Balancing helping and individual work – complex problem (Excerpt 1).

In the further course of interaction, the alternative explanatory accounts provided by the two students – i.e., capitalization on the one hand and compound spelling on the other hand – are successively processed and verified and an explanation to the question “why is there a line underneath it” (line 013) is co-constructed in permanent mutual inclusion and constant confirmation (lines 031–046). The fact that both participants devote their undivided attention to answering the questions shows that they consider the problems as both complex and important. After solving the problem, the interjected explanatory sequence is closed by a sign of thanks (Morek, 2012, p. 84f.). Yet again, the affective charge of this interaction becomes evident in Paula’s exuberant embrace. Finally, the students refocus again and solely on typing the letter (cf. Morek, 2015), so that the change in hierarchy and prioritization is reset. Figure 1 outlines how the two courses of action are intertwined and processed. The numbering of the respective black and blue arrows refers to the two competing courses of action.

The sequential analysis of this sequence reveals that the juggling of these two courses of action and the associated two moral commitments is anything but trivial. As can be seen at the sequential level, the individual assignment given by the teacher is generally prioritized over the request for help and the latter is suspended in favor of it. Nevertheless, this hierarchization and prioritization can also be reversed in the course of interaction, so that the help interaction is temporarily designated as the main activity. Moreover, it emerges that whenever the problem at hand is defined as complex by the students, the actual help, e.g., a collaborative explanation, requires extensive communicative effort. We now turn to the spatio-organizational dimensions of interaction and examine the embodied and spatial arrangements of the participants engaged in helping.

#### Spatial Organization

A prerequisite for engaging in helping is that the participants not only establish a shared focus of attention (Melander, 2009, p. 70) within a domain of scrutiny (Goodwin, 2003, p. 221) but also an interactional *space* for focused interaction. In addition, depending on how relevant and complex the participants define the problem and whether solving the problem also requires shared access to certain materials (screens, books, etc.), the interactional spaces are more or less stable and involve the use of various bodily resources. We illustrate how the way in which an interactional space is established reflects the moral order established by the participants and is also consequential for the constitution of the group as more or less transient.

As the teacher’s assignment for each student was to write an individual response letter on the laptop, the students are expected to orient their attention to their individual transactional segments; i.e., to the laptops in front of them (line 001: Paula, line 004: Tijen). These spatial segments are normally used exclusively by the person who creates them. Ciolek and Kendon (1980, p. 240) point out that “when people engage in writing, they do so within a small zone of space extending between themselves and a writing pad, and they will carefully maintain their exclusive access to this space.” In school, these segments are pre-designed for specific institutional purposes: learning-related activities. For this reason, we refer to them as being within the *individual space of task processing*. These individual spaces are embedded within the collective space of the classroom (Schmitt, 2013). To engage in help, participants need to direct their orientation away from their individual spaces and establish a shared space. In the present example, this takes three attempts.

Paula makes her first request for help (line 005) while looking at her screen (#1). When formulating the question (“is favorite subjects misspelled”; screen: “lieblings fächer”), she briefly turns her gaze to Tijen, yet her main orientation remains her text. Likewise, Tijen continues to gaze at her screen. Since both girls maintain their visual attention in their individual spaces of task processing, no shared space is established and the request remains unanswered.

After a short pause, Paula makes a second attempt to request help (line 007). This time, the two girls coordinate their detachment from their individual spaces by producing a summons (line 007) and a metadiscursive reply (line 008: “hang on”). Overlapping with this, Paula turns the notebook to Tijen (line 009) and begins to establish a “joint interaction space” (Ciolek and Kendon, 1980, p. 241); i.e., an overlap of their transactional segments. Her left hand remains on the laptop and delimits the dyad to the outside. Tijen’s visual orientation alternates between her own and Paula’s screen (line 11), with her left hand touching her own laptop. She responds to the repetition of the question (line 013: “why is there a line underneath it”) by shrugging her shoulders (line 014), thus indicating both a lack of knowledge and commitment to deal with Paula’s problem. Paula then reverses the establishment of a joint interaction space by moving the notebook back to its original position and returning to her individual space of task processing (line 017). By sighing, she expresses her regret, but at the same time, her acceptance that she has no right to receive help from Tijen. Shortly thereafter, Tijen also reverts to her individual space (line 020) and both girls continue working individually on their tasks for 16 s. Again, no group has been constituted and Paula’s problem remained unresolved.

Before initiating her third request for help, Paula indicates a detachment from her individual transactional segment by putting her hands together (line 025). After Paula’s attempted explanation (line 27: “is favorite subject maybe written as one word”), Tijen initially remains oriented toward her individual space and then turns her upper body toward Paula while displaying a change of state (Heritage, 1984) and this way announcing a solution (line 29: “I know why,” #9). Her pointing to Paula’s screen – i.e., the place where the problem is located – establishes a joint interactional space. Since both participants have the rights and responsibilities to perform actions that are relevant for the joint activity of helping within this space, we refer to it as the *shared space of helping*. Note that Tijens’s pointing is made possible by a rotation of her upper body. By doing so, she produces a “body torque,” (Schegloff, 1998), a posture that is characterized by “divergent orientations of body sectors above and below the neck and waist, respectively” (ibid., p. 536). This postural configuration is not only used to display dual involvements, but also a specific hierarchy of these involvements. The orientation of the lower body parts usually indicates which of the two concurrent activities is prioritized and going to be continued. The fact that Tijen only rotates her head and shoulders at first indicates a merely temporary involvement (line 029). Then, while proposing a solution (line 031), she rests her elbow next to her laptop and her head on her hand (#10), rotating the whole upper body inward and establishing a “stable-for-now home position” (Schegloff, 1998, p. 563), indicating a temporary but intense moral commitment to the help interaction. By turning away from her individual transactional segment, she demonstrates that she now gives priority to solving Paula’s problem and temporarily puts her own task on hold. Tijen indicates that her commitment to Paula’s problem is of a temporary nature by maintaining the orientation of her lower body to her individual space. The shared space of helping is therefore constructed as transient and fragile.

Paula checks Tijen’s proposal by putting the noun marker in front of the noun (line 033) and then implementing the suggestion (line 035; screen: “Lieblings fächer”). As the problem persists, as indicated by the fact that the text is still underlined, Tijen moves closer. When Paula proposes another solution, compound spelling (line 039), Tijen moves further forward, now also aligning her lower body with the shared space of helping and resting her head on her right hand (#11). With this thinking posture (Heller, 2021) and the full alignment of her body, she indicates that her orientation to the shared space of helping has assumed dominance and that she has fully committed herself to the search for a solution. At the same time, the bodily orientation along with the material arrangement makes the girls clearly identifiable as a group. Their intercorporeal arrangement shelters their interaction from being interfered with (cf. Ciolek and Kendon, 1980, p. 245) and enables an in-depth examination of the problem. Nevertheless, Paula’s request to wait (line 043) until the proposal has proven to be successful shows that from the point of view of the person seeking help, helping is a fragile concurrent activity and always runs the risk of being abandoned prematurely.

After the implementation of the suggestion leads to a solution of the problem (screen: “Lieblingsfächer”), Paula puts her arm around Tijen’s back (#12) so that they both sit shoulder to shoulder, staying visually oriented to the jointly mastered problem – the spelling, which is now error-free. The successful help therefore culminates in a tactile engagement through which both girls establish a high degree of closeness and withness (Ciolek and Kendon, 1980). The help interaction is then closed by both girls successively turning back to their individual spaces (lines 048–051).

In summary, the shared space of helping is established and constantly shaped through summons, response cries, requests, material arrangements, and bodily resources. With reference to the latter, body torque was shown to be a particularly flexible resource. Depending on how many body parts rotate and whether individual body parts such as the elbow assume a fixed position in the joint interactional segment, the shared space is arranged as more or less stable. The most stability is achieved through a full bodily alignment that also includes the lower body. All in all, the arrangement of the screens and the turning of the bodies away from the surrounding classroom shields the girls from the others present and visibly constitutes them as a group. It can be observed that in the more stable formations, there is deeper reflection on the spelling problem, which manifests in the extended explanatory activity. Especially in the classroom, a place with a large number of participants pursuing diverse courses of action, an arrangement such as this one seems functional in order to be able to focus one’s own attention on the problem and to signal to the outside that temporarily, no other attempts at interaction are desired. In addition to these cognitive and organizational functions, the closed formation of the shared space of helping is also associated with an affective charging of the interaction. The hug (Goodwin, 2017) as an intertwining motion of the body enabled mutual perception of tactile intimacy, affection, and withness. Together with the expression of gratitude, it worked as a “tie sign” (Goffman, 1971, p. 188–237) that was used to “affirm and support the social relationship between doer and recipient” (ibid., p. 63) and to build social trust between group members.

As various linguistic studies have already illustrated above, the complexity of the interactive elicitation of help interactions can vary to a great extent. While the example analyzed in this section reveals that the processing of a complex problem can entail considerable communicative effort, the opposite will be shown in the next sequence, in which the students interactively define the problem at hand as uncomplex.

### Helping in Handling a Problem Defined by the Students as Uncomplex

Unlike Excerpt 1, this sequence (Excerpt 2) does not take place entirely during the individual work phase, but during the transition from individual work to a teacher-led plenary activity. The students Marco, Karsten, Niko, Niclas, Robert, and Normen are sitting at a group table and are working on different tasks in which they learn how to write formal and informal letters.

**EXCERPT 2:**
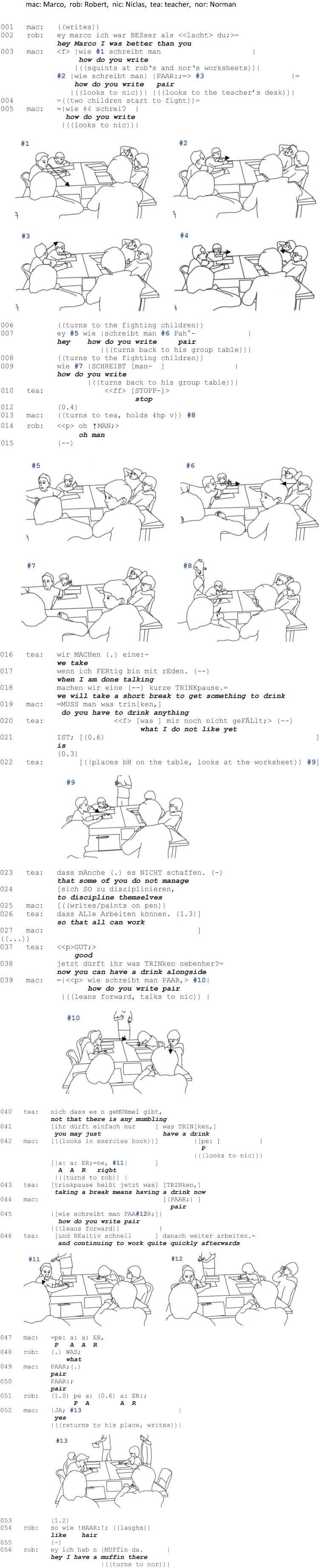
3DU_120905_11.

While Marco is writing his letter, he is confronted with a spelling problem and seeks help at his group table to solve this problem. During his first initiation attempts, still within the individual work phase, a dispute and disturbances break out in the class, prompting the teacher to issue an admonition directed at the entire class. Thus, in this sequence too, the students are required to balance two courses of action and two moral commitments. However, in this case, the second commitment does not (mainly) consist of the task to be done individually, but in following and sharing the plenary activity initiated by the teacher.

Again, we will first reconstruct the sequential organization and then focus on bodily and spatial dimensions.

#### Sequential Organization

While Marco continues writing after fooling around with Niko and Niclas, the other students are engaged in activities of their own: Karsten and Normen are working on their assignments, Niko and Niclas are conversing about the cameras set up in the classroom, and Robert is following this conversation.

In the immediate process of detachment from his own task, Marco realizes a first help request in the form of a request for information: “how do you write how do you write pair (line 003). Note that here, this question is not addressed to any of the students in particular. While asking this question with a raised voice, Marco eyes Robert and Normen’s papers, then turns his gaze to Niclas, who sits opposite to him but is looking to the front of the classroom, and finally looks himself to the teacher’s desk. In line 004, Marco attempts to re-engage the others again (Szymanski, 1999) and tries to establish eye contact with Niclas. Due to a disturbance caused by the dispute between two students in the back of the class, which attracts Marco’s attention, he abandons his turn in the middle and turns to the disputing children. It is striking that Marco brings forth his question again while still facing the fighting students and only gradually turns to his addressees; this time he incorporates the interjection “hey” (line 007) as “an ‘attention-getting’ device” (Schegloff, 1968, p. 70). Since Marco still fails to initiate a help interaction, he repeats the information question a fourth time, but cuts off again immediately after the teacher intervenes to stop the commotion (lines 008f.).

So far, there are two aspects that become evident: First, Marco’s verbal and embodied behavior (note that he is the person seeking help) shows that his attention is divided and he does not entirely devote his attention to the help process. Accordingly, he establishes a rather weak moral commitment to attending to his problem. At the same time, by instantly suspending his help request after the teacher intervenes in the students’ dispute, he prioritizes the teacher-led activity. This is also evident in the further course of interaction. Therefore, Marco postpones his problem throughout the teacher’s talk and only takes it up again when, in line 037, the teacher signals the conclusion of his admonition by the discourse marker “good” which indicates relaxation in the tense situation, and subsequently transitions into a short break (line 036). Again, Marco does not gain the attention of his addressees and does not succeed at recruiting a helper right away (line 037), so he then modifies his help request by repeatedly placing a candidate answer (Pomerantz, 1988) for disposal, which basically only requires ratification (lines 042 and 047) (ibid., p. 366). In line 048, Robert finally responds to Marco’s request, initiating a repair to get access to the problem at hand. Marco then only states the term his spelling problem revolves around (line 049f.) and Robert spells it out. With Marco’s confirmation of the spelling in line 050, which functions as a “sequence closing third (SCT)” (Schegloff, 2007, p. 118) and ends the question-answer-sequence, Marco finally revalues his epistemic status, which was initially devalued due to the help request. Marco detaches himself from the help interaction and returns to the main activity, i.e., writing the letter (line 052), although the teacher has announced a break. After a short delay, Robert adds a mnemonic rhyme in the form of a comparison (line 054), but Marco does not visibly take note of it.

**FIGURE 2:**
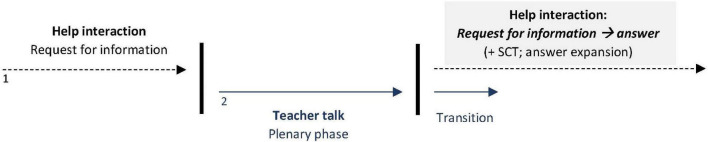
Balancing helping and plenary activities (Excerpt 2).

Recapping the sequential organization, it is apparent that the problem to be solved is not only not prioritized compared to the teacher’s activity, but was also defined as being of minor complexity and to be dealt with incidentally. As depicted in Figure 2, at its core, the help process comprises a simple question-answer adjacency pair sequence (Schegloff, 2007, p. 78) that is expanded with a third element (confirmation as a SCT), and an expansion of the helper’s answer (mnemonic rhyme). Furthermore, the help process is woven around the teacher’s activity so that the parallel courses of interaction do not seem to compete with each other to a considerable extent. Because of this clear prioritization and hierarchization of the different courses of action and the problem definition as a simple problem that can be solved later, and – most importantly – incidentally, the difficulty of balancing the two moral commitments does not arise to the same extent that it does as in Excerpt 1.

#### Spatial Organization

Considering the embodied and spatial arrangements the students make, it is apparent that Excerpt 2 not only differs with regard to the sequential organization from the first example, but also varies in the extent to which an interactional space is established and a group is constituted. Unlike in Excerpt 1, the shared space of helping assumes an open formation. This is usually accompanied by the fact that the group is also constituted as a transient association.

Since the request for help is made parallel to an announcement from and admonition by the teacher, the students direct their attention to the collective space. As already described above, Marco does not establish eye contact with a specific student when making his first request for help; while formulating the question (line 003), he gazes at his neighbors’ worksheets (#1), then to Niclas (without establishing a mutual gaze, #2), and finally to the teacher’s desk (#3). Twice, he abandons a repetition of the question while turning around to watch the fighting children at a different group table (lines 005–006, 007–008); a third time when the teacher calls “stop” (lines 009–010). Marco’s visual attention is oriented neither to his individual transactional segment nor to the classmates sitting at his group table, but outward to other children and the teacher, who walks from the back of the classroom to the front, projecting an upcoming plenary phase.

When the teacher reaches his desk in the front of the classroom, Marco performs a two-handed Vertical Palm Open Hand Prone gesture (Kendon, 2004, p. 252f.), which is directed to his classmates at his group table (#8). The gesture suggests that a course of action is interrupted. Simultaneously, Marco performs a body torque by turning his head and gaze toward the teacher, establishing a hierarchy between two concurrent activities: The direction of his head indicates his momentary orientation to the teacher in the collective space, while the direction of his lower body segments, which are still aligned with the group table, indicate that the group activity is the one to be continued. The division of resources – gaze vs. body posture and orientation – serve to keep both spaces, the collective and the potential shared space, present in the participants’ attention. In the present case, however, no shared space had been established beforehand. With the gesture, however, it is retroactively implied and the expectation is established for the classmates that they enter into a focused activity with Marco after the end of the teacher’s announcement.

During the teacher’s announcement (lines 016–036), Marco puts down his hands and begins to draw on his page (#9). His attention is no longer focused on the collective space, but on his individual transactional segment. Temporally coordinated with the closing of the teacher’s admonition, Marco addresses his fifth request for help to Niclas, who, however, is looking in a different direction (#10). While formulating the sixth request for help as a candidate answer, Marco turns to Robert (line 042). In doing so, he turns his head, leaving his hands in his individual transactional segment (#11), thus embodying his adherence to his individual space of task processing. A repetition of Marco’s request for help (lines 044–045) is accompanied by leaning his upper body forward into Robert’s transactional segment (#12). By approaching Robert’s individual space, an overlapping transactional segment, i.e., a potential shared space of helping, is established. However, the joint behavioral space is characterized by the fact that only one participant has bodily and visual access to the problematic spelling, because the worksheet remains in Marco’s individual transactional segment. Furthermore, the shared space is achieved unilaterally: Robert does not lean forward and even puts his head back a bit. In doing so, Robert assumes a body position from which he can simultaneously look at Marco and keep the teacher in view. Hence, Robert displays no dominant orientation toward Marco. As a consequence of Marco’s half turn and Robert’s forward-facing body, no closed formation emerges (Ciolek and Kendon, 1980) and the brief association of the two boys is not externally recognizable as a group, which minimizes the risk of being admonished by the teacher for not paying attention. Nevertheless, it can be observed that Robert takes up the request for help by indicating an acoustic comprehension problem (line 048). This allows him to spell out the word himself (line 051), therefore claiming more knowledge or a higher epistemic status (Heritage, 2012; Melander, 2012; Heller, 2018). Simultaneously, with a brief confirmation (line 052), Marco dissolves the body torque and with it the shared space of helping and returns to his individual space of task processing. This also dissolves the group.

Several aspects are worth mentioning here: Similar to example 1, multiple requests for help were necessary. Unlike in example 1, they had to be reconciled with the moral expectation to focus one’s attention on the teacher. The fact that several students did not respond to Marco’s request for help documents that, from the students’ perspectives, the moral commitment to follow the teacher’s activity was a priority. Another difference from example 1 is that the shared space here was established unilaterally and was characterized by an open formation. Parallel to the orientation toward the shared space, both boys maintained their orientation toward the individual space of task processing (Marco) or teacher (Robert). Thus, they were not recognizable as a group to outsiders. Overall, little effort was put into establishing the shared space: No material was moved, nor did any of the participants change their sitting position. The only resources that were used entailed repeated verbal requests and candidate answers, gaze, reducing distance, and body torque (limited to head rotation). This comparatively low effort in establishing the shared space enabled its rapid resolution when the help interaction was closed. It is also reflected with regard to the sequential organization, since at its essence, the help interaction merely comprises a canonical adjacency pair. Again, sequential as well as bodily spatial dimensions are closely linked to each other.

For a quick handling of the problem, a spatial arrangement such as the one used by Marco and Robert and their use of resources seems quite functional. Note, however, that the arrangement of the tables and the formations that emerge from them also influences the participants’ arrangements. While an I-shaped F-formation (Excerpt 1) (Ciolek and Kendon, 1980, p. 249) basically facilitates the establishment of a shared space of helping, in the case of an L-shaped F-formation, (Excerpt 2) (ibid., p. 249) this requires more (physical) effort. Finally, another difference to Excerpt 1 is that helping was not accompanied by affective engagement. Instead, Robert’s information about the spelling of the word only received a brief confirmation.

### Summary

The analyses have shown that students helping each other while pursuing classroom tasks have to balance two parallel, sometimes competing courses of action. They do this by hierarchizing these courses of action, prioritizing one activity over the other, and giving them space to varying degrees, both literally and figuratively speaking. In this process, it is crucial whether the problem being negotiated is interactively defined as either more complex or less complex. Depending on the complexity, the interactive negotiation requires more or less space. On a sequential level, this space is given through the provision or withholding of conversational space; in terms of the bodily spatial organization, a shared space of helping is created where all participants have equal access and the right to perform actions that are relevant for the joint activity of helping. Therefore, the extent to which the balancing of two moral commitments poses a problem is reflected in the embodied and spatial arrangements the students make. The more complex the students define the problem and the more they prioritize helping over the individual or plenary task, the more they engage with each other.

**FIGURE 3:**
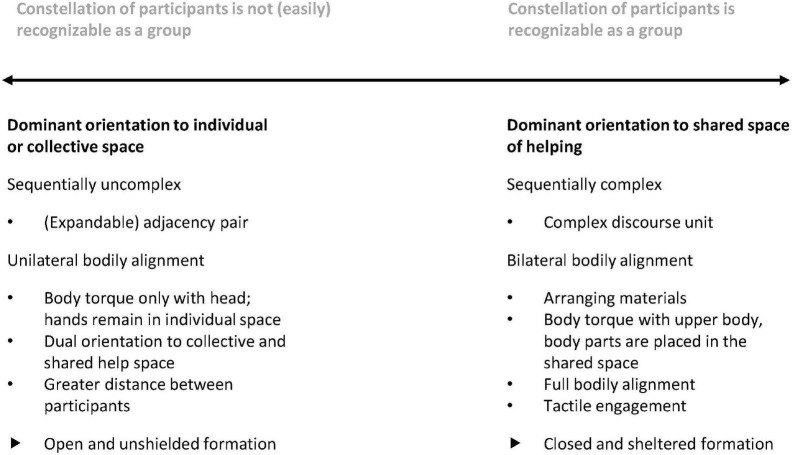
Dominant orientations within help interactions: A continuum.

We argue that the differences uncovered by our systematic comparison of the two prototypical sequences represent ends of a continuum between different dominant orientations. One end of the continuum is formed by dominance of the orientation to the shared space of helping, while the other end is formed by dominance of the orientation to the individual or collective space (cf. Figure 3). Analysis of our overall data set shows that help interactions are located between the ends of this continuum and differ with regard to which resources are used. Note that the two organizational forms are not related to the setting (individual work vs. plenary activities), but rather the interactively negotiated problem definition and compatibility of helping with the ongoing activity determine which form is established. Thus, even in the former setting, i.e., during individual work, we find rather short and less elaborate interactions that are more likely to be assigned to the right pole. The continuum further reveals that even within a single interaction, participants’ orientation constantly moves between these two ends of the continuum.

The intensity of the interaction and the way in which a shared space of helping is established is consequential for the constitution of the group. The more both interactants orient their visual and bodily orientation toward the shared space of helping and shield themselves from the outside, and the more conversational space they provide, the more they are recognizable as a group. The joint orientation to a shared space of helping also documents their moral commitment to find a solution and a high degree of withness.

## Discussion

Based on conceptualization of spontaneous helping in the classroom as a multiactivity (Haddington et al., 2014), the present study examined the different sequential and bodily spatial arrangements students adopt to manage dual involvements and, consequently, dual moral commitments (Raymond and Lerner, 2014): helping on the one hand and individual work or plenary activities on the other. Drawing on a video corpus comprising lessons in mathematics and German in two fifth-grade classes from different schools, the analyses revealed that helping can take various forms, ranging from very brief and seemingly low-effort to extended explanatory interactions. By conceptualizing helping as a multiactivity, it was shown that these different forms were not simply due to students’ motivation or willingness to help. Rather, the participants’ definition of the problem as more or less complex and important was a key moment for the constitution of these arrangements. Furthermore, the nature of the activity already underway, more specifically, the relative weight of the moral obligation associated with it played a crucial role. While the processing of an individual task was temporarily downgraded in its relevance or urgency (Excerpt 1), this was not the case with the teacher’s announcement to the class in Excerpt 2. In this case, the request for help was suspended until it no longer competed with the teacher’s activity. As a result, the way in which helping in the classroom is organized by students depends largely on how complex and relevant they define the problem at hand and whether they consider helping to be compatible with the ongoing activity.

We argue that the exemplary arrangements presented in this paper constitute a continuum, at the ends of which students either heavily commit to the help interaction and establish what we called a *shared space of helping* to which they temporarily orient themselves predominantly through bilateral bodily alignment, or momentarily insert helping into an ongoing course of action and remain primarily oriented toward their individual or a collective space (unilateral bodily alignment). In the first case, the students are publicly recognizable as a group due to their closed bodily-spatial formations (Ciolek and Kendon, 1980), the arrangement of relevant materials, and the duration of the interaction. In conjunction with the consideration of different settings, i.e., helping during an individual work phase or during teacher-led plenary activities, it also becomes evident that these groups are fragile and capable of dissolving, but also of forming at almost any time. In this context, Goffman (1963, 1964) dynamic notion of “engagement” proved central. It enabled us to show that participants’ engagement in helping continually oscillates between engagement and disengagement and is related to how they balance the moral obligations associated with the two activities.

Furthermore, we were able to show that the students use various practices to increase their fellow students’ moral obligation to provide help. Thus, they employ diverse affect displays, such as response cries, curses, and self-talk. Remarkably, these practices do not recruit help explicitly, but rather indirectly, by displaying the help-seeker’s affective experience of the problem. Using these resources, help-seekers establish only weak conditional relevancies and take into account the fact that they are not entitled to help if the other party is simultaneously pursuing another obligation.

With regard to quantitative research that models helping primarily as a cognitive activity and aims to optimize helping processes (Section “Helping in Educational Settings”) our qualitative approach has clarified that helping does not merely require certain cognitive steps, but rather perseverance in recruiting help as well as sophisticated interactive skills for balancing dual involvements and moral commitments. These requirements of helping-in-interaction are, in our view, important to keep in mind when systematically promoting helping between students. Thus, for promoting helping in the classroom, an important prerequisite is to provide students with opportunities and time for helping. Therefore, classroom activities would need to be arranged in such a way that helping can be better reconciled with competing tasks.

Finally, multimodal interaction analysis proved to be a productive approach with regard to methodologies used to study groups. Examining help interactions between students from the perspective of multimodal interaction analysis allowed us to highlight the moral intricacies involved in helping as a concurrent activity and to unpack the dynamics of engagement (Peräkylä et al., 2021) by describing the different sequential and bodily-spatial arrangements students created. Combining conversation analytic and context analytic concepts and procedures made it possible to reconstruct how participants coordinate both talk and bodily-spatial and material resources to create and orient to a shared space of helping and accomplish helping as a joint activity. Although we have analytically separated the description of the sequential and bodily-spatial organization of helping, they are interdependent and tightly intertwined (Goodwin, 2000). As a result, help interactions in which the participants are predominantly oriented toward the individual or collective space tend to be comparatively short, whereas those in which the orientation is dominantly toward the shared space of helping are sequentially more complex. Multimodal interaction analysis enables us to highlight precisely this interplay and to better understand the various ways in which groups are formed in interactive processes of helping.

## Data Availability Statement

The data analyzed in this study is subject to the following licenses/restrictions. The dataset presented in this article is not readily available because participants did not give written consent to share the data. Requests to access these datasets should be directed to VH, vheller@uni-wuppertal.de.

## Ethics Statement

Ethical review and approval was not required for the study on human participants in accordance with the local legislation and institutional requirements. Written informed consent to participate in this study was provided by the participants’ legal guardian/next of kin.

## Author Contributions

VH collected the data. DW identified and transcribed help sequences. DW and VH were equally involved in the development of the theoretical frame and the empirical analyses. Both authors contributed to the article and approved the submitted version.

## Conflict of Interest

The authors declare that the research was conducted in the absence of any commercial or financial relationships that could be construed as a potential conflict of interest.

## Publisher’s Note

All claims expressed in this article are solely those of the authors and do not necessarily represent those of their affiliated organizations, or those of the publisher, the editors and the reviewers. Any product that may be evaluated in this article, or claim that may be made by its manufacturer, is not guaranteed or endorsed by the publisher.

## Footnotes

1 After elementary school, the German school system is multi-tiered and distinguishes between Hauptschule, Realschule, Gymnasium (leading to a general qualification for university entrance), and various forms of comprehensive schools.
